# Compression of the Aorta in Uterine Hemorrhage

**Published:** 1852-01

**Authors:** 


*Compression of the Aorta in Uterine Hemorrhage.* By M. Chai. y-H nore.—M. Chailly-Honore considers that this practice is not resorted to so frequently as from its merits it deserves to be; and believes, that had it been employed in one or two cases in which transfusion has been lately performed, it would have rendered that *dernier resort* unnecessary, or would have enabled it to save life when employed. Rudiger employed compression so long back as 1797; but Ulsamer first advised its being applied through the wall of the abdomen in place of through the uterus. The practitioner, standing at the left side, passes his right hand between the uterus and intestines, seizes the vessel between the index and medius finger, fixing it firmly against the vertebral column, and pressing on his right with his left hand. If in thirteen cases in which this practice has been resorted to, half the women died, this arose from its being deferred until they were *in extremis*, and all other means had failed. To these cases M. Chailly opposes eighteen others, occurring in his own practice, and among which only one woman died, in whom also the application had been too long delayed. In some of these, compression was maintained for two hours without inconvenience. In the former series of cases the compression was delayed too long, and employed without rule, confidence, or patience. In the latter it was resorted to in time, and methodically continued. Of course the practice is not advocated as curative, but as a means of gaining time in an emergency, wherein time is everything.—*Brit. and For. Review, from Bull. de l' Academie.*

